# Impact of tumor size on survival of patients with resected pancreatic ductal adenocarcinoma: a systematic review and meta-analysis

**DOI:** 10.1186/s12885-018-4901-9

**Published:** 2018-10-16

**Authors:** Debang Li, Bin Hu, Yanming Zhou, Tao Wan, Xiaoying Si

**Affiliations:** 1 Department III of General Surgery, First Hospital of Lanzhou University, Lanzhou, China; 2 Department of Clinical Laboratory Medicine, First affiliated Hospital of Xiamen University, Xiamen, China; 3 Department of Hepatobiliary & Pancreatovascular Surgery, First affiliated Hospital of Xiamen University, Xiamen, China

**Keywords:** Pancreatic ductal adenocarcinoma, Resection, Size, Prognosis

## Abstract

**Background:**

The impact of tumor size on prognosis for surgically treated patients with pancreatic ductal adenocarcinoma (PDAC) remains controversial. A systematic review and meta-analysis was performed to evaluate this issue.

**Methods:**

Relevant studies published from January 2000 to June 2017 were identified through EMBASE and PUBMED. Data were pooled for meta-analysis using Review Manager 5.3.

**Results:**

Twenty eight observational studies involving a total of 23,945 patients were included. Tumors > 2 cm was associated with poor prognosis: the pooled hazard ratio (HR) estimate for overall survival was 1.52 (95% confidence interval [CI]: 1.41–1.64; *P* < 0.0001) by univariate analysis and 1.61 (95% CI: 1.35–1.91; *P* < 0.0001) by multivariate analysis; the pooled HR estimate for disease-free survival was 1.74 (95% CI: 1.46–2.07; *P* < 0.0001) by univariate analysis and 1.38 (95% CI: 1.12–1.68; *P* = 0.002) by multivariate analysis. When compared with patients with tumors ≤2 cm, those with the tumors > 2 cm had higher incidences of lymph node metastasis, poor tumor differentiation, lymph vessel invasion, vascular invasion, perineural invasion, and positive intraoperative peritoneal cytology.

**Conclusion:**

These data demonstrate that PDAC size > 2 cm is an independent predictive factor for poor prognosis after surgical resection and associated with more aggressive tumor biology.

## Background

Pancreatic ductal adenocarcinoma (PDAC) represents 90% of pancreatic cancers and is the fifth leading cause of cancer-related death in Western countries. Complete surgical resection is the only option that can offer hope of prolonged survival; however, the long-term survival remains unsatisfactory with a 5-year survival rate around 20% because of the high frequency of postoperative disease recurrence [1]. Therefore, it is necessary to identify prognostic factors to help stratify patients for appropriate management categories. Tumor specific factors, such as the margin status, histological differentiation, lymph node metastasis, and vascular invasion, have been shown to predict poor clinical outcomes [2, 3]. Tumor size is also a significant prognostic factor and is included in tumor node metastasis system (TNM) classification. According to the American Joint Committee on Cancer (AJCC) staging system for PDAC, the optimum tumor size cutoff value distinguishing T1 and T2 disease is 2 cm [4]. Despite the availability of many publications, the impact of PDAC size on prognosis remains controversial [5, 6]. A systematic review and meta-analysis of the literature was therefore undertaken to investigate this issue.

## Methods

### Study selection

The present study was performed by following the recommendations of the Preferred Reporting Items for Systematic Reviews and Meta-Analyses (PRISMA) Statement [7]. An electronic search of the PUBMED and EMBASE databases from January 2000 to June 2017 were performed to identify relevant citations. The following keywords were used: “pancreatic cancer”, “pancreatic ductal adenocarcinoma”, and “prognosis”. The reference lists of all retrieved articles were manually reviewed in order to identify additional studies.

### Criteria for inclusion and exclusion

All original full-text articles reporting the impact of tumor size using a cut-off of 2 cm on overall survival (OS) or disease-free survival (DFS) in patients with PDAC after resection were considered eligible. Abstracts, letters, editorials and expert opinions, reviews without original data, case reports, non-human studies, non-English language studies, studies using values of cut-off other than 2 cm for tumor size, and studies that included other periampullary carcinomas (ampullary, duodenal, and biliary) in the same study cohort without separate assessments were excluded.

### Data extraction and methodological assessment

All selected studies were evaluated independently by two investigators (ZY and SX) for data extraction and quality assessment. Disagreement in the evaluation of studies was resolved by discussion and consensus. Parameters extracted included first author, study origin, year of publication, study design, type of resection, pathology, available long-term outcomes, and univariate and multivariate hazard ratios (HR) for OS and DFS.

The level of evidence of each study was categorized according to the Evidence-Based Medicine Levels of Evidence [8].

### Statistical methods

Data for OS and DFS were analyzed using HR with 95% confidence intervals (CI), and a HR >1 represents a worse outcome. Between-study heterogeneity was assessed with I^2^ statistics, and a value of > 50% was considered significant heterogeneity. A funnel plot based on the OS outcome was conducted to evaluate the presence of publication bias. The differences in clinicopathologic features were estimated as a pooled odds ratio (OR) with 95% CI. All analyses were performed using the Review Manager 5.3 (Cochrane Collaboration, Software Update, Oxford). A value of *P* < 0.05 was considered statistically significant.

## Results

### Selection of studies

**Fig. 1:**
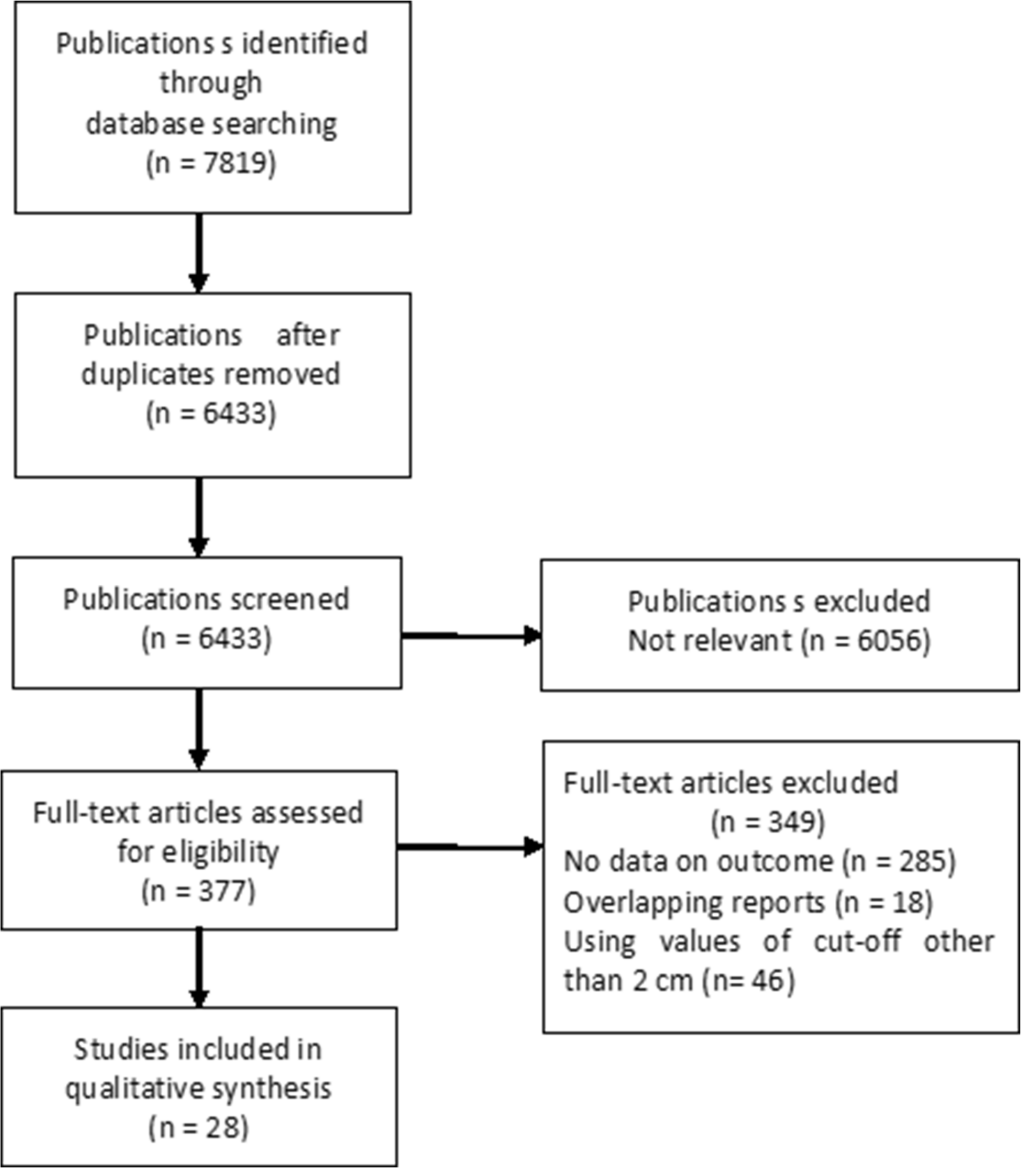
Flowchart of study selection

A total of 28 studies comprising 23,945 individuals were identified for inclusion (Fig. 1). The summary characteristics of the included studies are shown in Table 1 [5, 6, 9–34]. There were no randomised controlled trials (RCT). All these studies were observational in nature and classified as level-4 evidence. There were 18 single-center [5, 9, 10, 12, 13, 15, 17, 19–21, 23–28, 32, 33] and 10 multicenter studies [6, 11, 14, 16, 18, 22, 29–31, 34].

**Table 1:** The main characteristics of included studies

Reference	Year	Country	N	TS > 2.0 cm, n (%)	TRPD/DP/TP	R0 R, n (%)	LNM, n (%)	PNI, n (%)	PTD, n (%)	MOS (Months)	5-yr OS (%)
Meyer [9]	2000	Germany	91	67/86 (77.9)	−/−/−	93 (100)	66 (72.5)	41 (45.1)	14 (16.3)	16.8	10.5
Ahmad [10]	2001	USA	116	70/94 (74.4)	−/−/−	88 (75.8)	73 (62.9)	–	61 (52.5)	16	19
Kim [11]	2006	USA	70	50 (71.4)	68/2/0	–	40 (57.1)	46 (65.7)	26 (37.1)	21	19
Smith [12]	2008	UK	109	81 (74.3)	109/0/0	80 (73.3)	88 (80.7)	–	36 (33.0)	13.9	–
Chiang [13]	2009	Taiwan,	159	123 (77.3)	−/−/−	114 (71.6)	95 (59.7)	–	32 (20.1)	–	12.5
Chang [14]	2009	Australia	365	281 (76.9)	295/70/0	233 (63.8)	217 (59.5)	256 (70.1)	98 (26.8)	16.8	11.4
Kato [15]	2009	Japan	176	148 (84.1)	176/0/0	115 (65.3)	123 (69.8)	145 (82.3)	11 (6.2)	9.9	12.3
Massucco [16]	2009	Italy	77	60 (77.9)	63/0/14	59 (76.6)	59 (76.6)	58 (75.3)	50 (64.9)	16.5	–
Bhatti [17]	2010	UK	84	78 (92.8)	84/0/0	49 (58.3)	56 (66.6)	–	24 (28.5)	22	13
de Jong [5]	2011	USA	1697	1279 (75.4)	1640/0/57	1213 (71.8)	1280 (75.4)	1126 (66.3)	649 (38.2)	18.3	21.2
Cannon [18]	2012	USA	245	213 (86.9)	220/20/0	184 (75.1)	–	–	72 (29.4)	18.3	–
Petermann [19]	2013	Switzerland	86	76 (88.3)	86/0/0	89 (68.6)	72 (83.7)	–	–	16.8	–
Yamada [20]	2013	Japan	390	312 (80.0)	288/71/31	–	277 (71.0)	–	–	–	–
Buc [21]	2014	France,	306	–	242/45/19	195 (72.5)	214 (71.3)	212 (83.8)	–	34	32
Elberm [22]	2015	UK	1070	–	1070/0/0	482 (45.9)	757 (70.7)	–	–	18.5	–
Iwagami [23]	2015	Japan	39	27 (69.2)	−/−/−	–	14 (35.9)	34 (87.2)	3 (7.6)	–	–
Liu [24]	2015	USA	411	242 (58.9)	411/0/0	379 (92.2)	223 (54.3%)	–	150 (36.5)	–	–
Okumura [25]	2015	Japan	230	–	155/66/9	190 (82.6)	135 (58.7).	–	33 (14.3)	–	–
Yamamoto [26]	2015	Japan	195	156 (80.0)	123/61/11	138 (70.7)	145 (74.3)	108 (55.3)	–	27.1	34.5
Lin [27]	2016	China	233	189 (81.1)	233/0/0	196 (84.1)	161 (69.1)	–	147 (63.1)	–	19.0
Abe [28]	2017	Japan	355	273 (76.9)	215/98/22	282 (79.4)	223 (62.8)	282 (79.4)	137 (38.5)	–	–
Ansari [29]	2017	USA	15,398	12,725 (82.6)	−/−/−	–	–	–	–	–	16.1
Chikamoto [30]	2017	Japan	138	66 (47.8)	138/0/0	–	46 (33.3)	–	10 (7.2)	–	–
Marchegiani [6]	2017	Italy, USA	1507	1183 (78.5)	1179/268/59	840 (55.7)	1149 (76.2)	1376 (91.3)	468 (31.1)	26.0	–
Kurata [31]	2017	Japan	90	41 (45.6)	−/−/−	–	31 (34.4)	–	–	–	–
Le [32]	2017	USA	93	70/86 (81.3)	93/0/0	–	78 (84.7)	–	50 (53.8)	40.6	–
Watanabe [33]	2017	Japan	122	98 (87.5)	73/47/2	122 (100)	62 (55.3)	–	6 (4.9)	21	27
Yu [34]	2017	China	93	32 (34.4)	−/−/−	89 (96.6)	49 (52.6)	52 (55.9)	36 (38.7)	–	–

*UK* United Kingdom, *PNI* peri-neural invasion, *TS* tumor size, *LNM* lymph node metastasis, *PTD* poor tumor differentiation, *MOS* median overall survival, *TR* type of resection, *PD* pancreaticoduodenectomy, *DP* distal pancreatectomy, *TP* total pancreatectomy, *R0 R* R0 resection

### Meta-analysis

**Fig. 2:**
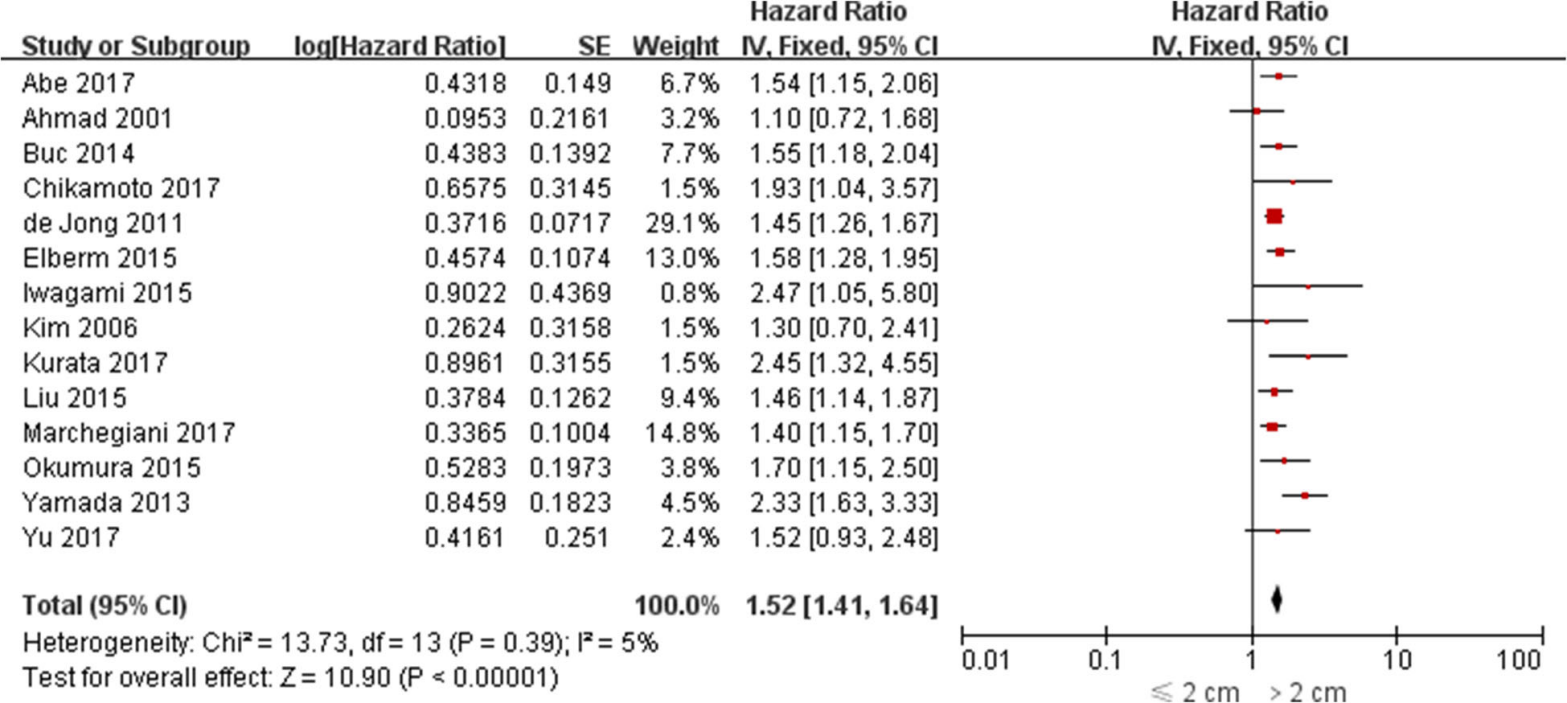
Forest plots for univariate meta-analysis of overall survival

**Fig. 3:**
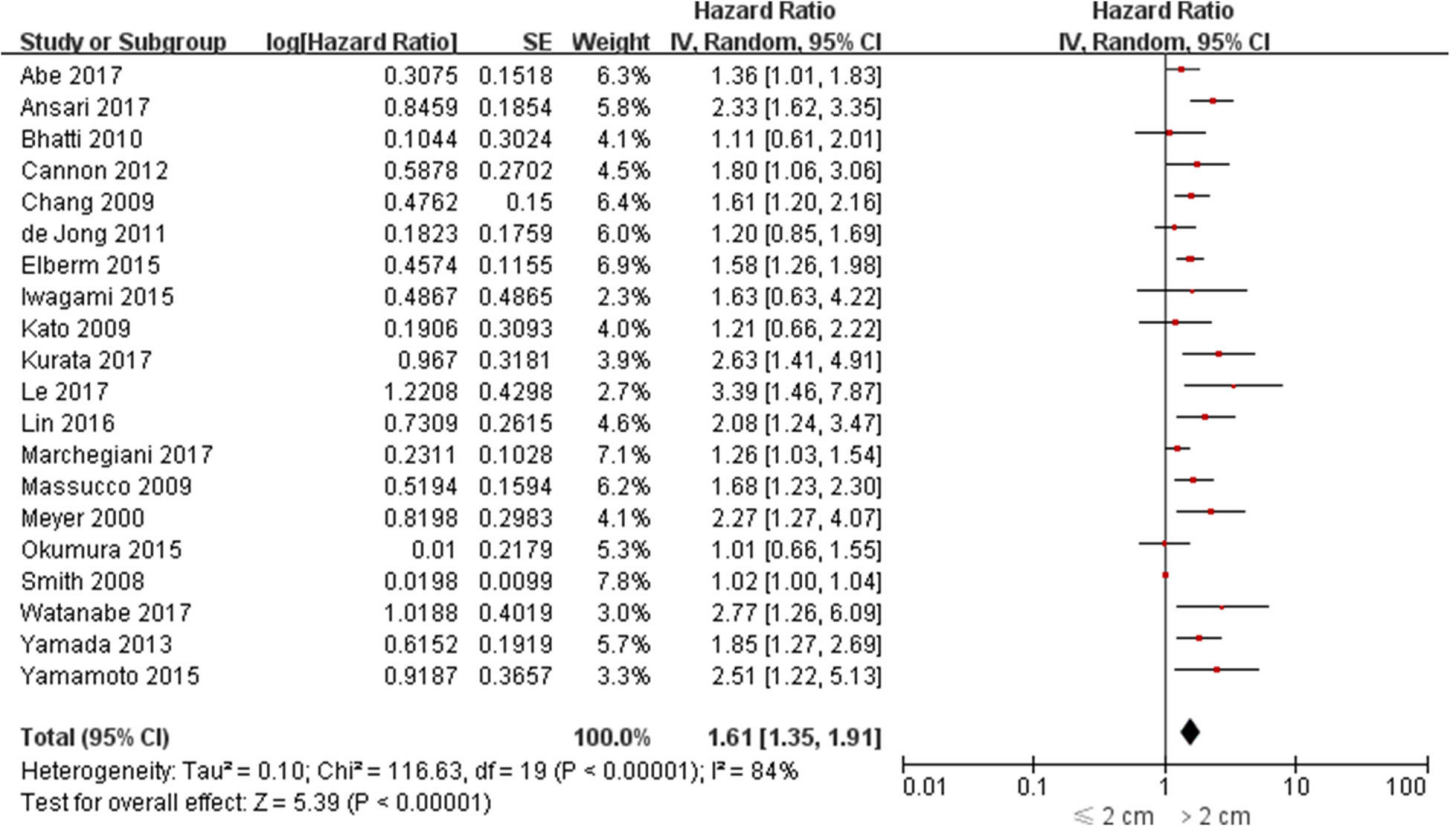
Forest plots for multivariate meta-analysis of overall survival

The impact of PDAC size on OS was evaluated in 26 studies [5, 6, 9–13, 15–18, 20–34], among which univariate HR was reported in 14 [5, 6, 10, 11, 21–25, 28, 30, 31, 34] and multivariate HR was reported in 20 [5, 6, 12, 14–18, 20, 22, 23, 25–29, 31–33]. Both univariate and multivariate HR were reported in 8 studies [5, 6, 20, 22, 23, 25, 28, 31]. The pooled HR estimate for OS was 1.52 (95% CI: 1.41–1.64; *P* < 0.0001) by univariate analysis and 1.61 (95% CI: 1.35–1.91; *P* < 0.0001) by multivariate analysis (Figs. 2-3). In sensitivity analysis, exclusion of any single study from the analysis did not alter the results significantly (data not shown). Also, the results from three subgroup analysis were in line with those from overall analyses (Table 2).

**Table 2:** Subgroup analysis for the influence of tumor size on overall survival after pancreatic ductal adenocarcinoma resection

Subgroup	No. of studies	HR	95% CI	*P*-value	I^2^ (%)
Single centre studies					
Univariate analysis	8	1.52	1.39, 1.67	< 0.001	29
Multivariate analysis	13	1.53	1.22, 1.91	< 0.001	76
Multicentre studies					
Univariate analysis	7	1.54	1.36, 1.74	< 0.001	0
Multivariate analysis	7	1.67	1.41, 1.99	< 0.001	51
Western studies					
Univariate analysis	8	1.46	1.34, 1.59	< 0.001	0
Multivariate analysis	11	1.55	1.25, 1.92	< 0.001	87
Eastern studies					
Univariate analysis	7	1.82	1.55, 2.15	< 0.001	0
Multivariate analysis	10	1.62	1.40, 1.87	< 0.001	35

*CI* confidence interval, *HR* hazard ratio

**Fig. 4:**
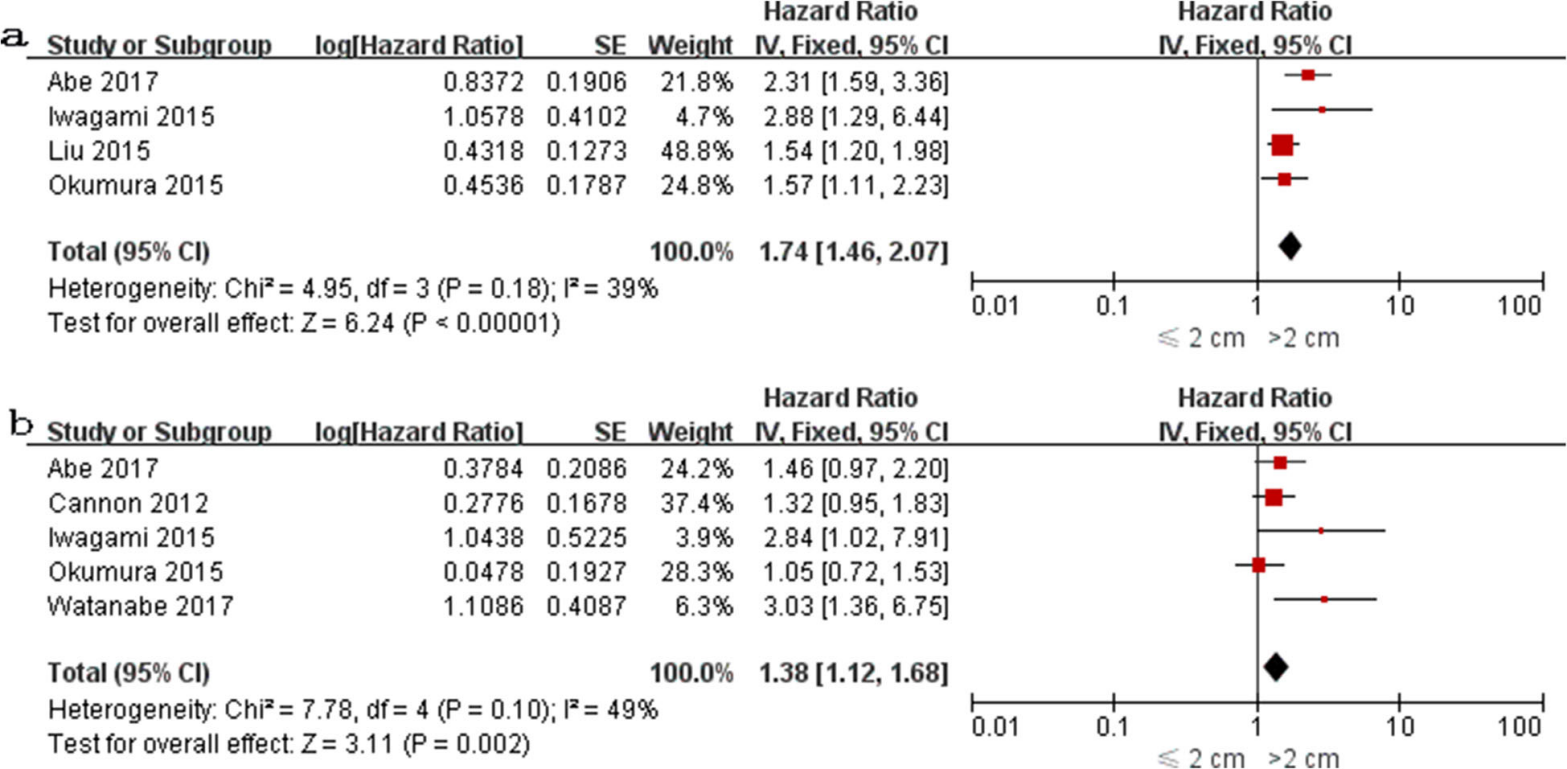
Forest plots for univariate (**a**) and multivariate (**b**) meta-analysis of disease-free survival

The impact of PDAC size on DFS was evaluated in 6 studies [18, 23–25, 28, 33], among which univariate HR was reported in 4 [23–25, 27] and multivariate HR was reported in 5 [18, 23, 25, 28, 33]. Both univariate and multivariate HR were reported in 3 studies [23, 25, 28]. The pooled HR estimate for DFS was 1.74 (95% CI: 1.46–2.07; *P* < 0.0001) by univariate analysis and 1.38 (95% CI: 1.12–1.68; *P* = 0.002) by multivariate analysis (Fig. 4a-b). Sensitivity and subgroup analyses were not performed due to the small number of studies.

Nine studies compared the clinicopathological factors between tumors > 2 cm and tumors ≤2 cm groups [5, 6, 9, 13, 15, 19, 20, 23, 28]. Pooled analysis showed that patients with tumor > 2 cm had higher incidences of lymph node metastasis (79.1% vs. 64.2%, OR 2.24, 95% CI: 1.43–3.51; *P* < 0.001), poor tumor differentiation (36.2% vs. 28.4%, OR 1.45, 95% CI: 1.22–1.73; *P* < 0.001), perineural invasion (80.8% vs. 67.1%, OR 1.89, 95% CI: 1.22–2.92; *P* = 0.004), vascular invasion (39.8% vs. 27.7%, OR 1.78, 95% CI: 1.41–2.24; *P* < 0.001), positive resection margins (36.9% vs. 27.2%, OR 1.56, 95% CI: 1.31–1.87; *P* < 0.001), and positive intraoperative peritoneal cytology (14.2% vs. 2.6%, OR 5.66, 95% CI: 2.15–14.93; *P* < 0.001), as compared with patients with tumors ≤2 cm.

### Publication bias

**Fig. 5:**
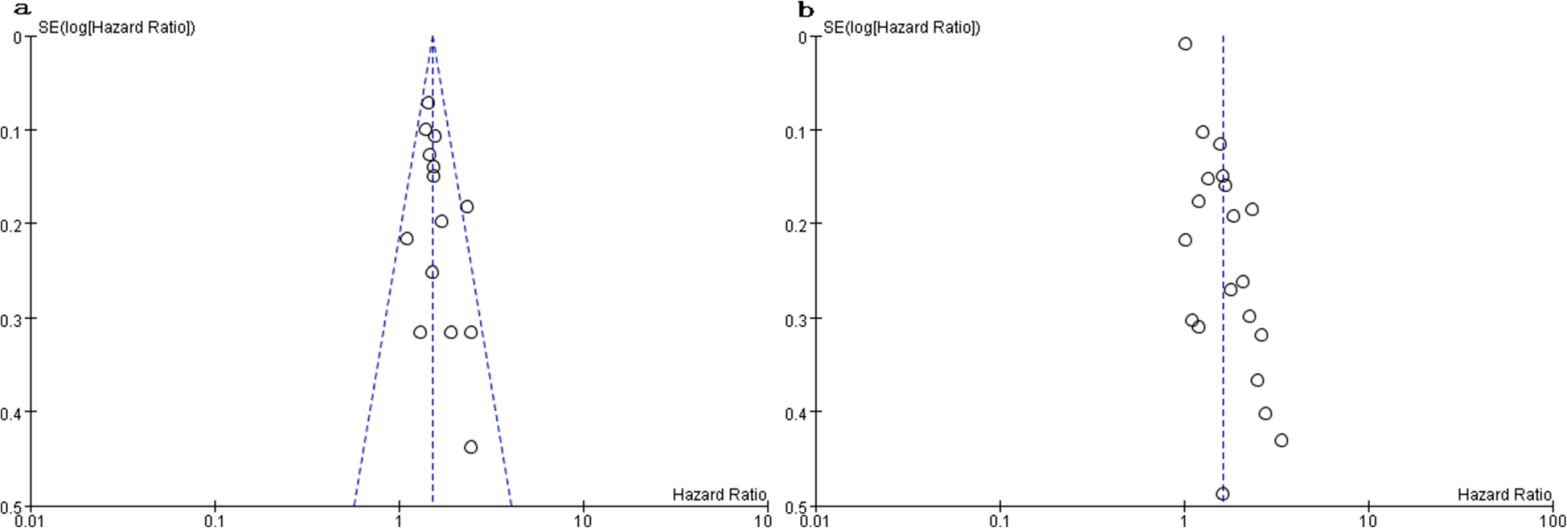
Analysis of publication bias in meta-analysis of univariate (**a**) and multivariate (**b**) overall survival

No significant funnel plot asymmetry was observed in the meta-analysis of univariate and multivariate OS (Fig. 5a-b).

## Discussion

Assessment of tumor size for prognostication had better reproducibility for both clinical and pathologic staging [35]. Indeed, many studies investigating the prognostic factors in PDAC have shown that tumor size is one of the most important parameters in predicting the clinical outcome of cancer patients. The cut-off point for PDAC size in the published reports varies from 2, 2.5, 3, 4, and 5 cm [6]. Generally, tumors ≤2 cm in the greatest dimension are defined as small PDAC [36]. Some authors noted that tumors > 2 cm have prognostic implications after resection [6, 12, 14, 16], while others failed to confirm this finding [5, 10, 11]. Meta-analysis provides a way to increase statistical power and resolves inconsistencies. Our pooling data have shown that tumors > 2 cm have negative impact on the survival of patients with PDAC. These findings affirm the validity of the T-stage of the current AJCC classification, in which the cut-off value of 2 cm is proposed to be the sole factor determining whether a pancreatic tumor is staged as T1 or T2 disease [4]. When the clinicopathologic findings in the two groups were compared, patients with tumors > 2 cm showed higher incidences of lymph node metastasis, poor tumor differentiation, lymph vessel invasion, vascular invasion, perineural invasion, positive resection margin, and positive intraoperative peritoneal cytology, implying that tumors > 2 cm intrinsically have more aggressive tumor biology that contributes to worse prognosis. Marchegiani et al. speculated that tumor size could be considered a surrogate of neoplastic progression, knowing that it is an expression of time passing from its original development. Therefore, a tumor with bigger dimensions often implies a relatively delayed diagnosis and therefore has a higher likelihood of being associated with other adverse pathologic factors [6].

The PDAC size also has impact on operative outcomes. Patients with tumors > 2 cm were found to be associated with more intra-operative blood loss and a greater need for packed red blood cell transfusion [5], knowing that the latter variable may lead to worse oncologic outcomes via transfusion-related immune modulation [37].

There is growing evidence that neoadjuvant therapy is associated with a statistically significant reduction in the tumor positive margin status, tumor stage and grade, lymph node metastasis, and perineural invasion, thereby resulting in improved survival in patients with initially resectable PDAC [38]. However, identification of patients who will benefit from neoadjuvant therapy remains challenging. Unlike other malignant pathological features of PDAC, tumor size can be diagnosed by preoperative imaging and therefore may be able to guide clinical decision making. Our results show that tumors > 2 cm are characterized by the presence of other relevant poor prognostic factors and therefore can be considered as an indication for neoadjuvant therapy. The potential aim is to achieve dual purposes of attenuating malignant pathological features on the one hand and improving the surgical outcome on the other. Randomized controlled trials are necessary to confirm this preliminary recommendation.

This review is limited by the low quality. All included studies were retrospective in nature and classified as level-4 evidence, which underlines the validity of the analyzed outcomes. Ansari et al. [29] found that the association between survival and PDAC size was linear in patients with localized tumors but stochastic in patients with regional and distant stages. Unfortunately, none of the included studies analysed the stage-dependent relationship between PDAC size and survival. Similarly, subgroup analysis based on anatomic location of the PDAC could not be performed due to insufficient data.

The strength of our findings is that it represents a variety of clinical settings, including Eastern and Western data rather than the sole experience of a single institution. In addition, these pooled results based on multivariate analysis do not differ essentially from those of analyses based on univariate analysis. These findings indicate that tumors > 2 cm, per se rather than a confounder, have a prognostic implication. Finally, there is no evidence of publication bias.

## Conclusion

The current evidence demonstrates that PDAC size > 2 cm is an independent predictive factor for poor prognosis after surgical resection and associated with more aggressive tumor biology. Future trials are necessary to evaluate the survival benefit of neoadjuvant therapy in this subset of patients.

## Abbreviations

95% CI: 95% confidence interval
DFS: Disease-free survival
DM: Diabetes mellitus
HR: Hazard ratio
OR: Odds ratio
OS: Overall survival
PDAC: Pancreatic ductal adenocarcinoma
PRISMA: Preferred reporting items for systematic reviews and meta-analyses
WMD: Weighted mean difference
